# Harnessing “little mighty” cockroaches: Pest management and beneficial utilization

**DOI:** 10.1016/j.xinn.2023.100531

**Published:** 2023-10-20

**Authors:** Chonghua Ren, Nan Chen, Sheng Li

**Affiliations:** 1 Guangdong Provincial Key Laboratory of Insect Developmental Biology and Applied Technology, Guangzhou Key Laboratory of Insect Development Regulation and Application Research, Institute of Insect Science and Technology & School of Life Sciences, South China Normal University, Guangzhou 510631, China; 2 Guangdong Laboratory for Lingnan Modern Agriculture, Guangzhou 510631, China; 3 Guangmeiyuan R&D Center, Guangdong Provincial Key Laboratory of Insect Developmental Biology and Applied Technology, South China Normal University, Meizhou 514779, China

## Rationale backs up cockroaches being “little mighty”

**Figure 1:**
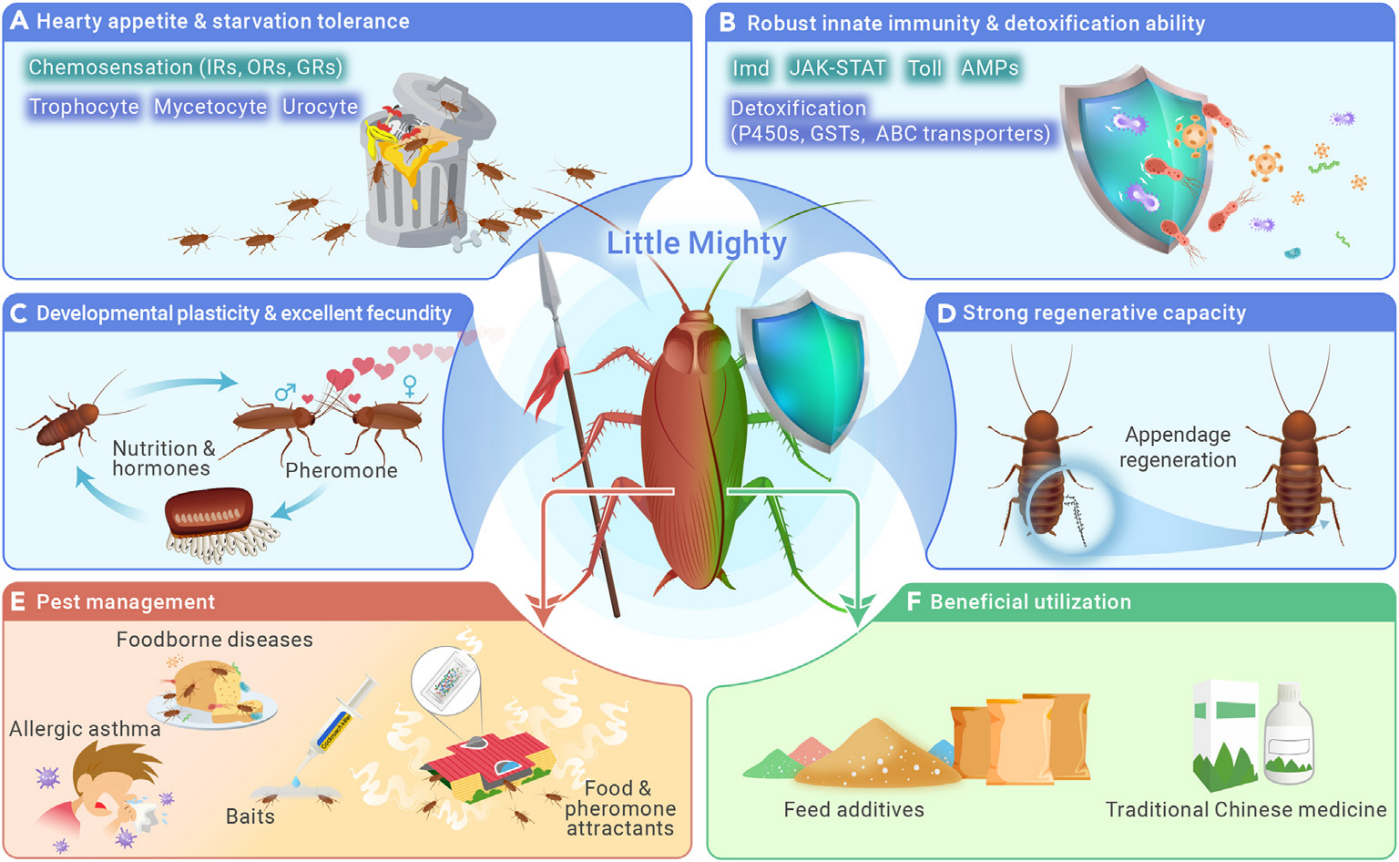
Harnessing “little mighty” cockroaches: pest management and beneficial use (A–D) Four biological traits, including a hearty appetite and starvation tolerance (A), robust innate immunity and detoxification ability (B), developmental plasticity and excellent fecundity (C), and strong regenerative capacity (D), collectively underpin cockroaches’ reputation as “little mighty.” Cockroaches display a combination of a spear (urban pests, in red) and a shield (beneficial insects, in green). (E) Cockroaches are culprits in human allergies and foodborne diseases (left), and bait and eco-friendly strategies can be used for efficient cockroach control (right). (F) Beneficial use of cockroach-derived products in feed additives and traditional Chinese medicine.

Cockroaches belong to the insect order Blattodea and often serve as model organisms for hemimetabolous insects. They occupy a pivotal evolutionary position in studies of insect metamorphosis. The emergence of the last common ancestor of all extant cockroaches may date back to approximately 235 million years ago. Cockroaches are a highly successful group of insects: about 5,000 living species have been recorded. Since the seismic continental drift during the ancient transition from Pangaea, cockroaches have undergone substantial invasion and distribution to all continents worldwide.^1^ The American cockroach (*Periplaneta americana*) and the German cockroach (*Blattella germanica*) are the most common and are closely synanthropic species, referred to as “little mighty” (a household name, *xiao qiang*, in China) because of their extreme vitality and adaptation. The rationale behind cockroaches’ being considered “little mighty” can be attributed mostly to four biological characteristics (Figure 1).

### Hearty appetite and starvation tolerance

Most cockroach species are not picky about food, and they are renowned for their omnivorous diet. Both *P. americana* and *B. germanica* have evolved a remarkable chemoreception system for recognizing edible substrates. Multiple types of chemoreceptors, including olfactory receptors (ORs), ionotropic receptors (IRs), and especially gustatory receptors (GRs), exhibit a remarkable expansion, surpassing that of any other insect species with sequenced genomes.^2^^,^^3^ They also possess a powerful detoxification system for tolerating bitter tastes and toxic substances, which enhances their food diversity.^3^ Regarding starvation tolerance, cockroaches can survive for several weeks in the absence of food resources. In addition to the regular trophocyte, in which stored glycogen and lipids can be mobilized during the early stage of starvation, cockroaches have two other cell types in the fat body: urocytes and mycetocytes. In response to severe and extensive starvation, urocytes release stored uric acids, which are subsequently recycled to produce amino acids in mycetocytes, and these amino acids serve as an additional energy resource during starvation (Figure 1A).

### Robust innate immunity and detoxification ability

Cockroaches inhabit primarily moist and unsanitary environments, frequently encountering moldy foods and various microbes and pathogens. In response to viral, bacterial, and fungal infections, the Imd, JAK-STAT, and Toll pathways are activated, resulting in the production of antimicrobial peptides (AMPs). These AMPs in the hemolymph efficiently eliminate invading microorganisms. Cockroaches exhibit extensive gene duplication of innate immunity components, especially Gram-negative binding proteins and AMPs, resulting in significantly stronger immunity. Likewise, their detoxification ability is facilitated by the expansion of metabolic enzymes, including cytochrome P450s, glutathione transferases (GSTs), and ATP-binding cassette (ABC) transporters (Figure 1B).^3^

### Developmental plasticity and excellent fecundity

Hemimetabolous cockroaches undergo several nymphal instars that prepare them for subsequent metamorphosis. These developmental processes are controlled predominantly by nutritional and hormonal signals.^3^^,^^4^ In particular, IIS-TORC1 and 20E coordinate final body size by controlling growth rate and developmental timing, respectively. Meanwhile, JH signaling antagonizes 20E-induced metamorphosis, therefore maintaining the nymphal status. Consequently, cockroaches exhibit elaborately nutrition- and hormone-coordinated developmental plasticity, especially in the American cockroach.^3^

Gregarious cockroaches usually exhibit large populations, largely because of their excellent fecundity. An adult cockroach can produce hundreds of progeny that hatch from oothecae, which serve as protective barriers against various xenobiotics and external injury, leading to an extremely high survival rate of newborns. Sex-pheromone-based chemical communication is crucial for courtship, mating, and consequently reproduction. Sex differentiation and JH signaling coordinate sex-specific and high attractiveness in mature females of the German cockroach, ensuring that courtship occurs only between the opposite sexes.^5^ Notably, the American cockroach is capable of facultative parthenogenesis when male partners are not available, producing solely female progeny without paternal genetic contribution. However, parthenogenesis does not occur in the German cockroach (Figure 1C).

### Strong regenerative capacity

Cockroaches are among the top-ranked insects in terms of regenerative capacity and have been used for the establishment of several regenerative models, such as the polar coordinate model and the boundary model. Cockroach regeneration involves both internal tissues and external appendages. In terms of internal tissues, the neuropile and commissures of the brain, as well as the meta- or mesothoracic ganglia, display remarkable reforming ability after ablation. The endocrinal prothoracic glands can regenerate after artificial extirpation as well. Regarding external appendages, all types of nymphal appendages can regenerate to some degree following damage. The legs, in particular, can fully regenerate to their original morphology after a single molting,^3^ and meanwhile, the internal tissues, including muscles, nerves, and the trachea, can be well restored (Figure 1D).

Altogether, these four characteristics dominantly contribute to remarkable vitality and environmental adaptation, underpinning cockroaches as “little mighty.” These characteristics have facilitated the use of cockroaches as an ideal hemimetabolous insect model for studying developmental biology, evolutionary biology, immunology, toxicology, chemical ecology, reproductive physiology, and regenerative biology. Several key scientific questions in cockroach biology are worthy of in-depth study, including the following: (1) How do chemical communication systems coordinate complex sexuality? (2) What factors initiate facultative parthenogenesis in the American cockroach? (3) How do cockroaches possess such a robust regenerative capacity? Moreover, a long-standing challenge lies in understanding the driving forces behind cockroach-termite evolution and the origin of eusociality in termites.

## Cockroach management and beneficial use

Dialectically, some cockroach species are regarded as both serious unban pests and as beneficial insects. With a holistic understanding of the underlying mechanisms behind “little mighty,” a solid foundation can be established for pest management and beneficial use of this group of insects.

### Urban pest management

The German cockroach and American cockroach are strictly synanthropic urban pests. They infest almost all human-built structures, harbor and transmit a variety of pathogens, and produce and emit nearly twenty protein substances that trigger asthma and allergic diseases. Cockroach control is at a critical point worldwide as pesticide resistance becomes increasingly troublesome. Currently, the most widely used commercial baits and spray formulations strongly rely on chemical pesticides. These chemistry-based strategies intensify the resistance issue, becoming increasingly unacceptable from an eco-friendly perspective. Meanwhile, cockroach baits are far from sufficient regarding their attractiveness. Given that cockroaches are omnivorous and excellent chemical communicators with typical aggregative and sexual behaviors, pheromone-based behavioral regulation along with physical trapping offers a highly specific and eco-friendly strategy for cockroach management (Figure 1E). Notably, in recent years, some parasitic wasps, green muscardine fungus, and densovirus have been shown to be promising tools for biological control of certain cockroach species, and nanoparticle-based techniques may also be worth exploring in the future.

### Beneficial insect use

Cockroaches provide various ecological, economic, medical, and biomimetic benefits. In terrestrial ecosystems, they serve as both a food source and a predator in the food chain. Some species digest plant matter and convert it into a form that can be easily decomposed by microorganisms. In China, cockroaches are even used directly for the disposal of food waste in industry. Economically, given their strong antimicrobial activity, cockroach extracts can be used as valuable feed additives to improve the health of livestock and aquaculture. Cockroach extracts have also been used in cosmetics and daily chemical products. In terms of medical use, cockroaches hold significant value in traditional Chinese medicine (TCM), which might be attributable to their potential association with a strong regenerative capacity. This use was recorded in ancient Chinese medical encyclopedias such as *Shennong Ben Cao Jing* and *Compendium of Materia Medica*. Modern prescribed drugs derived from alcohol extracts of the American cockroach*,* such as the well-known “Kangfuxin solution,” have been extensively used in treating gastrointestinal ulcers and chronic heart failure (Figure 1F). Regarding bionics, the unique characteristics of cockroaches significantly inspire advancements in robotics and cyborg technology.

In short, their remarkable vitality and strong adaptive ability make cockroaches a combination of a spear (urban pests) and a shield (beneficial insects) (Figure 1). Thus, it is urgent to harness both strengths to control and/or use these fascinating creatures. From a public health perspective, characterizing cockroach allergens and elucidating the inner mechanism will receive more attention in clinical medicine. Developing food and sex and/or aggression pheromone attractants with physics adhesion should be a promising direction for cockroach management. Meanwhile, the active components of cockroach-derived TCM remain undefined, and elucidation of the regeneration mechanism in cockroaches will provide a theoretical foundation for identification of active ingredients and for cockroach-derived TCM.

## Conclusion

A long-standing challenge lies in how to harmoniously harness the “little mighty” cockroaches: efficiently controlling cockroach pest populations and making the best use of cockroach resources for human beings. By closing these gaps with fundamental research, we aim to gain a deeper appreciation of the complex nature of cockroaches and thus provide novel insights into pest management and beneficial use.
